# Assessment of microbiota present on a Portuguese historical stone convent using high‐throughput sequencing approaches

**DOI:** 10.1002/mbo3.1030

**Published:** 2020-04-30

**Authors:** Tânia Rosado, Luís Dias, Mónica Lança, Carla Nogueira, Rita Santos, Maria Rosário Martins, António Candeias, José Mirão, Ana Teresa Caldeira

**Affiliations:** ^1^ HERCULES Laboratory Évora University Évora Portugal; ^2^ Chemistry Department School of Sciences and Technology Évora University Évora Portugal; ^3^ Geosciences Department School of Sciences and Technology Évora University Évora Portugal

**Keywords:** biocolonization, biodegradation/biodeterioration, biodeteriogenic activity, metagenomic DNA, microbiota assessment, stone material biodecay

## Abstract

The study performed on the stone materials from the Convent of Christ revealed the presence of a complex microbial ecosystem, emphasizing the determinant role of microorganisms on the biodecay of this built cultural heritage. In this case study, the presence of *Rubrobacter* sp., *Arthrobacter* sp., *Roseomonas* sp., *and Marinobacter* sp. seems to be responsible for colored stains and biofilm formation while *Ulocladium* sp., *Cladosporium* sp., and *Dirina* sp. may be related to structural damages. The implementation of high‐throughput sequencing approaches on the Convent of Christ's biodecay assessment allowed us to explore, compare, and characterize the microbial communities, overcoming the limitations of culture‐dependent techniques, which only identify the cultivable population. The application of these different tools and insights gave us a panoramic view of the microbiota thriving on the Convent of Christ and signalize the main biodeteriogenic agents acting on the biodecay of stone materials. This finding highlighted the importance of performing metagenomic studies due to the improvements and the reduced amount of sample DNA needed, promoting a deeper and more detailed knowledge of the microbiota present on these dynamic repositories that support microbial life. This will further enable us to perform prospective studies in quarry and applied stone context, monitoring biogenic and nonbiogenic agents, and also to define long‐term mitigation strategies to prevent biodegradation/biodeterioration processes.

## Graphical abstract

A multianalytical approach based on culture‐dependent methods, high‐throughput sequencing, and scanning electron microscopy was implemented to characterize the microbiota on stone of the Convent of Christ (Tomar, Portugal). The biocolonizer agents detected induce aesthetic damages and structural alterations on these materials, promoting the appearance of stains, biofilms, and detachment of stone fragments. These findings are a starting point for the implementation of a long‐term monitoring plan to prevent the decay of this monument and promote its safeguard.

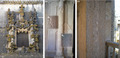

## 1 INTRODUCTION

The identification of microorganisms on historical objects traditionally involves classical microbiology techniques. Typical cultivation‐based methods enable quantitative and biochemical activity analyses of isolated strains. However, these microbiology approaches highly limit the investigation of microbial biodiversity occurring in the environment. Unfortunately, a low percentage of microorganisms can be cultivated by standard techniques and the cultivable microorganisms are under‐represented considering the microbial diversity present in the Earth (González & Saiz‐Jiménez, 2005; Sanchez‐Moral et al., 2005). Despite the limitations inherent to this approach, culture‐based techniques and the development of new culture media are still encouraged due to the advantage of having pure cultures isolated to carry out physiological and metabolic studies (Dakal & Arora, 2012; Dyda et al., 2018).

Nevertheless, to understand the phenomena that promote the degradation of cultural assets it is crucial to obtain a deeper knowledge about the colonizing microbial population. In this way, techniques based on nucleic acids allow the differentiation of microorganisms within complex microbial communities or the identification of isolated microorganisms (Portillo & Gonzalez, 2009).

Therefore, DNA sequencing approaches are very useful to phylogenetic identification and have been applied in several areas, being suitable in artworks to analyze the microbial diversity (Cappitelli et al., 2009; Carmona et al., 2006; Olivares et al., 2013; Saarela et al., 2004; Schabereiter‐Gurtner, Piñar, Vybiral, Lubitz, & Rolleke, 2001).

Small subunit ribosomal DNA genes like 16S and 18S universally present in all prokaryotes and eukaryotes, respectively, provide an efficient mean to identify microorganisms. These ribosomal sequences possess variable and highly conserved regions, which are used as phylogenetic markers to identify and distinguish the different microorganisms on all phylogenetic levels (Dakal & Arora, 2012; Kennedy & Clipson, 2003; Li, Zhang, He, & Yang, 2016; Zhang, Ping, Zhou, Wang, & Zhang, 2018). The internal transcribed spacer (ITS) region is also an effective tool for the identification of eukaryotic population (Anderson & Cairney, 2004; Dakal & Arora, 2012; Kennedy & Clipson, 2003). In the case of yeast, the sequencing of the D1/D2 domain of 26S/28S rDNA region has been used to identify these microorganisms from different sources. This approach is rapid and precise when compared with the physiological method for the yeast identification and has also been applied to study the phylogeny of different yeast groups and species‐level differentiation (Dagar, Kumar, Mudgil, Singh, & Puniya, 2011; Hesham, Wambui, Ogola, & Maina, 2014; Kiyuna et al., 2012; Lv, Huang, Zhang, Rao, & Ni, 2013; Selbmann et al., 2014). Other nucleic acid approaches can be applied to detect uncultivable microorganisms. Recent developments in sequencing chemistries, bioinformatics, and automated instruments have revolutionized the knowledge of microbial diversity (Dyda et al., 2018; Edgar, Haas, Clemente, Quince, & Knight, 2011; England & Pettersson, 2005; Li et al., 2016; Mardis, 2008; Schubert, Lindgreen, & Orlando, 2016; Shendure & Ji, 2008; Zhang, Chiodini, Badr, & Zhang, 2011; Zhang et al., 2018; Zhou et al., 2015). The ongoing advances in genomics and sequencing technologies are allowing a *new era* of microbial community analyses using culture‐independent approaches such as metagenomics, metaproteomics, metatranscriptomics, and proteogenomics which are fundamental for the full identification of the microbial diversity and to understand their interactions with biotic and abiotic factors (Rastogi & Sani, 2011).

High‐throughput sequencing (HTS) technologies (also known as next generation sequencing—NGS) have revolutionized the study of microbial diversity. HTS is currently used in multidisciplinary fields in academic, clinical, and industrial settings, particularly to identify mammal species or to study microbial diversity in soils, freshwater, human guts, wastewater treatment facilities, and others (Jones et al., 2009; Nam, Jung, Roh, Kim, & Bae, 2011; Panek et al., 2018; Roh et al., 2009; Ronholm, 2018; Ye & Zhang, 2011; Zhang et al., 2018). This technology enables the characterization of microbial diversity but also allows a better knowledge about the functions, activities, and dynamics of microbial communities thriving in their natural environments (Zhou et al., 2015). In the last 4–5 years, the potentialities of this methodology have been explored in cultural heritage assets such as mural paintings (Rosado, Mirao, Candeias, & Caldeira, 2014), wood and brick materials (Gutarowska et al., 2015; Koziróg et al., 2014), carbonate stones (Chimienti et al., 2016; Dias et al., 2018), sandstone buildings (Cutler, Oliver, Viles, Ahmad, & Whiteley, 2013), parchments, and books (Kraková et al., 2018; Teasdale et al., 2015) in order to do the microbiota characterization (Perito & Cavalieri, 2018).

This work aims to encompass a comprehensive multidisciplinary approach to characterize the biological colonization and consequently biodeterioration/biodegradation of an architectural stone material (Ançã limestone) present in the Convent of Christ, a UNESCO World Heritage monument located in Tomar (Central Portugal). This emblematic Convent has been studied by our research group since 2015 (Rosado et al., 2016) owing to the visible evidence of alteration on the stone materials which are responsible for structural and aesthetic damages like stains, biofilm formation, delamination, and detachment of stone fragments.

In order to reach an answer for the degradation/deterioration processes that are affecting the stone, culture‐dependent methods (CDM), scanning electron microscopy (SEM), and HTS were applied in the multiple zones of the Convent showing different alteration processes to (a) characterize the microorganisms that are colonizing the stone surfaces and (b) evaluate the distribution patterns of the microbiota.

## 2 MATERIALS AND METHODS

### 2.1 Sampling process

The monumental complex of the Convent of Christ in Tomar (N39°36′14.8536″, W8°25′9.231″) is classified as UNESCO World Heritage monument, and it is an emblematic exemplar of the Portuguese history that possesses a wide diversity of sculpted stones with visible marks of biocontamination. This monument stands at the top of a hill in Tomar, a city with hot and dry summers, as well as cold winters, with precipitation and partially overcast sky. Temperatures often reach 38°C in the summer and −1°C in the winter. The yearly rainfall average is 773 mm.

**FIGURE 1:**
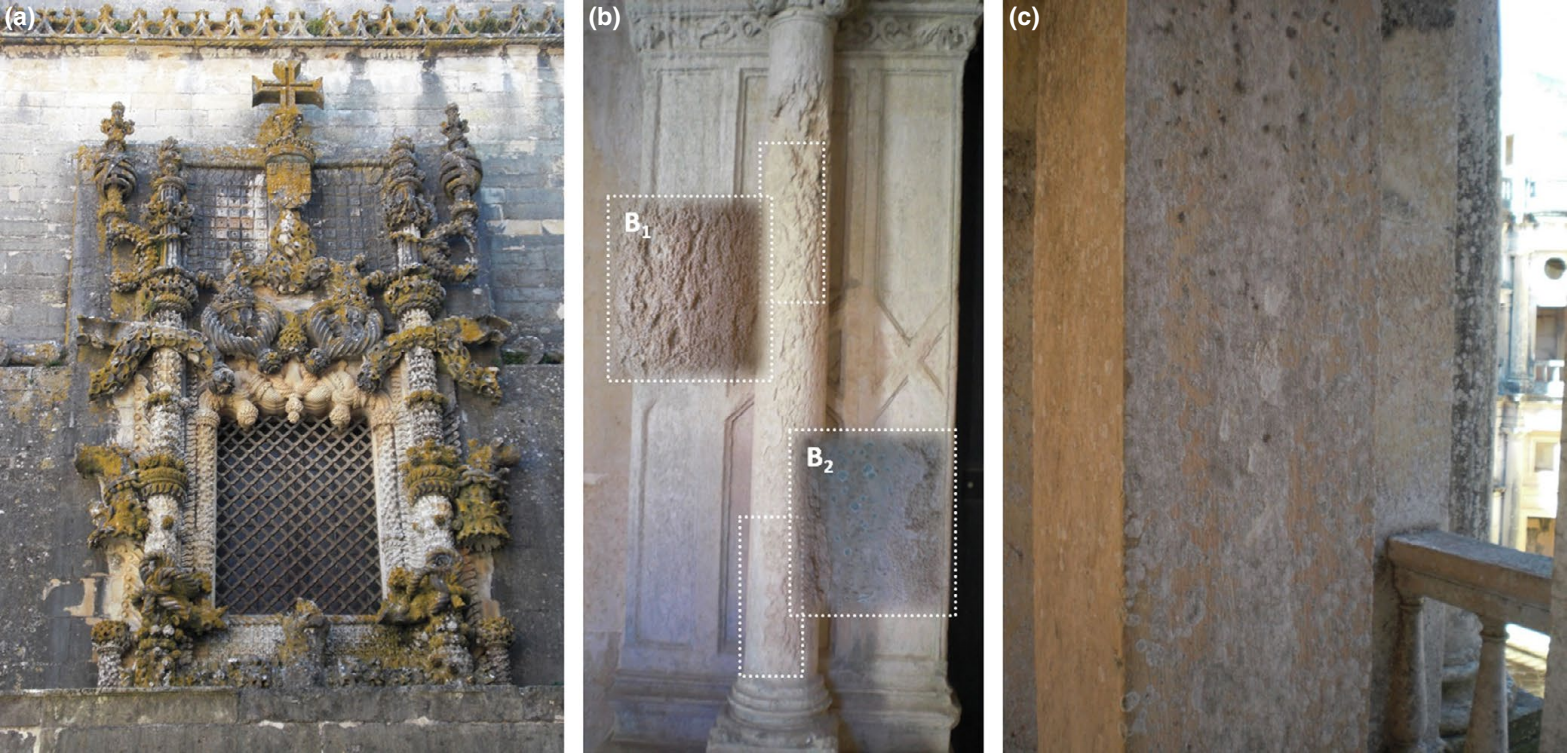
Chapter Window (a), Primitive Cloister (b), and 1st floor of the Main Cloister (c) from Convent of Christ whose biodecay was assessed

The sampling process was performed on representative areas of the stone materials present in the Convent of Christ, including the areas with evidence of alterations, under the coordination of a conservator‐restorer (Figure 1).

Microinvasive (chisel and scalpels) and noninvasive (swabs) methods were applied during the samples’ collection, under semi‐aseptic conditions (collection performed with sterile material but in outdoor environment). Microsamples of stone (10 samples with less than 1 mm^2^) were removed using a small chisel to perform the material characterization, close to losses or cracks in order to avoid further damage. For the microbiological assays, samples were also collected under semi‐aseptic conditions with sterile swabs and placed in a suspension of transport MRD medium (Maximum Recovery Diluent) until utilization.

### 2.2 Biocolonization assessment

To assess the deterioration degree of the support and the presence of biocolonization, microfragments of stone were used as such or coated with Au‐Pd (Balzers Union SCD 030) during 30 s, and observed in a HITACHI S‐3700N variable pressure scanning electron microscope (VP‐SEM) with an accelerating voltage of 10 kV. Microanalyses of the selected samples were performed using the same microscope coupled with a Bruker XFlash 5010 energy‐dispersive X‐ray spectrometer (EDX) to allow a microstructural characterization of the stones and to obtain the elemental composition (point analyses and 2D mapping). EDX analyses were performed at 20 kV.

### 2.3 Characterization of microbial communities

#### 2.3.1 Cultivable population

The samples collected for biological assays were mechanically shaken during 24 hr at 150 rpm. After this period, each sample was inoculated in solid cultures containing different culture media (Nutrient Agar—NA, Malt Extract Agar—MEA, Cooke Rose Bengal—CRB, ASM‐1 and BG‐11), selective for several kinds of microorganisms (bacteria, fungi, and cyanobacteria, respectively). Bacterial isolation procedures were carried out in Nutrient Agar, at 30°C and for 48 hr. For fungal colonies’ isolation, two standard mycological media (MEA and CRB) were used, and the cultures were incubated for 7 days at 28°C. For cyanobacterial development, the samples were inoculated in ASM‐1 and BG‐11 (solid and liquid media) and were incubated at room temperature (20–25°C) with periods of light exposure (16:8 hr light–dark cycle) for 1 month. After this period, the plates were maintained at the same temperature mentioned above to detect slow bacterial/fungal development.

The distinct single colonies obtained were subcultured onto NA/MEA Petri dishes and maintained on NA/MEA slants at 4°C.

The different microbiological strains were identified following the standard methods (Domsch, Gams, & Anderson, 1980), based on their macro‐ and micromorphological characteristics. For each bacterial/fungal isolate, the genes encoding the 16S/ITS sequence, respectively, were amplified by PCR, purified, and sequenced by outsourcing service (STAB VIDA). The nucleotide sequences were aligned with those retrieved from the GenBank (NCBI) databases for the homology analysis using the BLASTN 2.8.0 program.

#### 2.3.2 Biological communities

##### DNA extraction, HTS, and data processing

Metagenomic DNA was extracted from stone samples (0.1 g) using QIAmp DNA Stool Mini Kit (Qiagen), with slight modifications from the manufacturer's instructions.

Bacterial and fungal communities were characterized by Illumina Sequencing for the 16S rRNA V3‐V4 region and ITS 2, respectively.

DNA was amplified for the hypervariable regions with specific primers and further reamplified in a limited‐cycle PCR reaction to add sequencing adaptor and dual indexes. First, PCR reactions were performed for each sample using 2X KAPA HiFi HotStart Ready Mix. In a total volume of 25 μl, 12.5 ng of template DNA and 0.2 μM of each PCR primer were used.

For bacteria, the following primers were used: forward primer Bakt_341F 5′‐CCTACGGGNGGCWGCAG‐3′ and reverse primer Bakt_805R 5′‐GACTACHVGGGTATCTAATCC‐3′ (Herlemann et al., 2011; Klindworth et al., 2013). For fungi, a pool of forward primers was used: ITS3NGS1_F 5′‐CATCGATGAAGAACGCAG‐3′, ITS3NGS2_F 5′‐CAACGATGAAGAACGCAG‐3′, ITS3NGS3_F 5′‐CACCGATGAAGAACGCAG‐3′, ITS3NGS4_F 5′‐CATCGATGAAGAACGTAG‐3′, ITS3NGS5_F 5′‐CATCGATGAAGAACGTGG‐3′, and ITS3NGS10_F 5′‐CATCGATGAAGAACGCTG‐3′ with the reverse primer ITS3NGS001_R 5′‐TCCTSCGCTTATTGATATGC‐3′ (Tedersoo et al., 2014). The PCR conditions involved 3 min of denaturation at 95°C, followed by 25 cycles of 98°C for 20 s, 55°C for 30 s and 72°C for 30 s, and a final extension at 72°C for 5 min. Negative controls were included for all amplification reactions. Electrophoresis of the PCR products was undertaken on a 1% (w/v) agarose gel, and the ~490 bp V3–V4 and ~390 bp ITS2 amplified fragments were purified using AMPure XP beads (Agencourt, Beckman Coulter) according to the manufacturer's instructions. Second, PCR reactions added indexes and sequencing adaptors to both ends of the amplified target region by the use of 2X KAPA HotStart Ready Mix, 5 μl of each index (i7 and i5) (Nextera XT Index Kit, Illumina) and 5 μl of the first PCR product in a total volume of 50 μl. The PCR conditions involved a 3‐min denaturation at 95°C, followed by 8 cycles of 95°C for 30 s, 55°C for 30 s and 72°C for 30 s, and a final extension at 72°C for 5 min. The Amplicon PCR products were analyzed by electrophoresis agarose gel (1%, w/v), and the amplified fragments were purified using AMPure XP beads (Agencourt, Beckman Coulter) according to the manufacturer's instructions. The amplicons were quantified by fluorimetry with a Quantus Fluorometer ONE dsDNA quantitation kit (Invitrogen, Life Technologies), pooled at equimolar concentrations and paired‐end sequenced with the V3 chemistry in the MiSeq^®^ according to the manufacturer's instructions (Illumina) at Genoinseq. They were multiplexed automatically by the Miseq^®^ sequencer using the CASAVA package (Illumina) and quality‐filtered with PRINSEQ software (Schmieder & Edwards, 2011) using the following parameters: (a) bases with average quality lower than Q25 in a window of five bases were trimmed, and (b) reads with less than 220 bases were discarded for V3–V4 samples and less than 100 bases for ITS2 samples. The forward and reverse reads were then merged by overlapping paired‐end reads using the Adapter Removal v2.1.5 (Schubert et al., 2016) software with default parameters. The QIIME package v1.8.0 (Caporaso et al., 2010) was used for operational taxonomic unit (OTU) generation, taxonomic identification, sample diversity, and richness indexes’ calculation. Sample IDs were assigned to the merged reads and converted to fasta format (split_libraries_fastq.py, QIIME). Chimeric merged reads were detected and removed using UCHIME (Edgar et al., 2011) against the Greengenes v13.8 database (DeSantis et al., 2006) for V3–V4 samples and the UNITE/QIIME ITS v12.11 database (Abarenkov et al., 2010) for IST2 samples (script identify_chimeric_seqs.py, QIIME). OTUs were selected at 97% similarity threshold using the open reference strategy. First, merged reads were prefiltered by removing sequences with a similarity lower than 60% against the Greengenes v13.8 database for V3–V4 samples and the UNITE/QIIME ITS v12.11 database for ITS2 samples. The remaining merged reads were then clustered at 97% similarity against the same databases listed above. Merged reads that did not cluster in the previous step were again clustered into OTU at 97% similarity. OTUs with less than two reads were removed from the OTU table. A representative sequence of each OTU was then selected for taxonomy assignment (pick_rep_set.py, assign_taxonomy.py; QIIME).

## 3 RESULTS AND DISCUSSION

Preliminary studies performed on the built stone areas of the Convent of Christ revealed evident signals of biocontamination linked to some weathering effects, which is leading the stone to serious problems (Rosado et al., 2016). Alteration products like calcium oxalates and carotenoids were identified on deteriorated areas of the stone, which seems to be correlated with the presence of microbial contamination as a result of their metabolic activity (Rosado et al., 2016).

Thus, to be able to answer the key questions of the stone alteration processes, complementary methodologies based on culture‐dependent techniques, metagenomic approaches, and analytical methods were used to characterize the biocolonizers present on the stone building materials of the Convent of Christ. There are several advantages on the use of a multianalytical approach, which will be further presented and discussed in this paper.

As mentioned above, this monument exhibits formation of biofilms, stains, and structural degradation covering several outdoor areas (Figure 1). The surrounding environment (temperature, humidity, sun, and wind) together with the bioreceptivity of the stone materials seems to contribute to its biocolonization (Adamson, McCabe, Warke, McAllister, & Smith, 2013; Scheerer, Ortega‐Morales, & Gaylarde, 2009; Török & Přikryl, 2010).

The monitored areas present different pathologies. In the Chapter Window, it is possible to observe red, green, and black patinas (Figure 1a), while on the Primitive Cloister (PC) pink/orange color appearance (Figure 1b_1_), fungal proliferation (Figure 1b_2_), and structural damages (Figure 1b_1,2_) are visible on the column, changing its surface roughness. On the other hand, on the 1st floor of the Main Cloister (MC) white/greenish colored areas can be found together with biofilms covering the stone surfaces. Besides these aesthetic damages, structural alterations such as stone powdering are also present.

**FIGURE 2:**
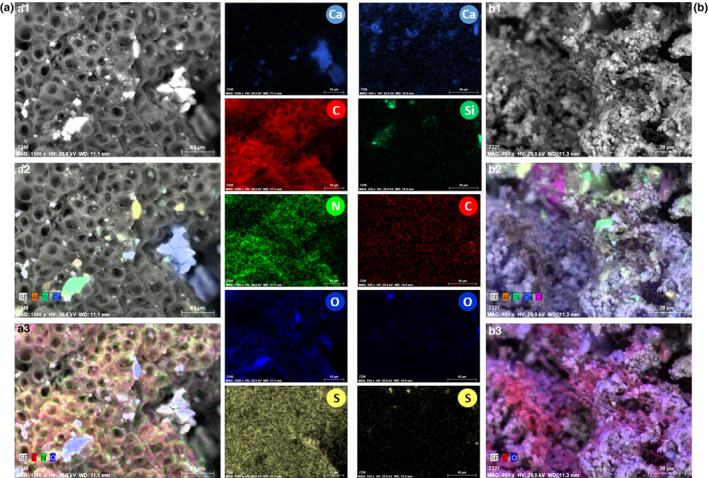
Analysis of stone microfragments from the Manueline Window (a) and the Main Cloister (b) of the Convent of Christ, by SEM observation in back‐scattered mode (a_1_ and b_1_) and EDX 2D elemental maps (a_2–3_ and b_2–3_) with individual element distribution of calcium (Ca), silicon (Si), carbon (C), nitrogen (N), oxygen (O), and sulfur (S)

**FIGURE 3:**
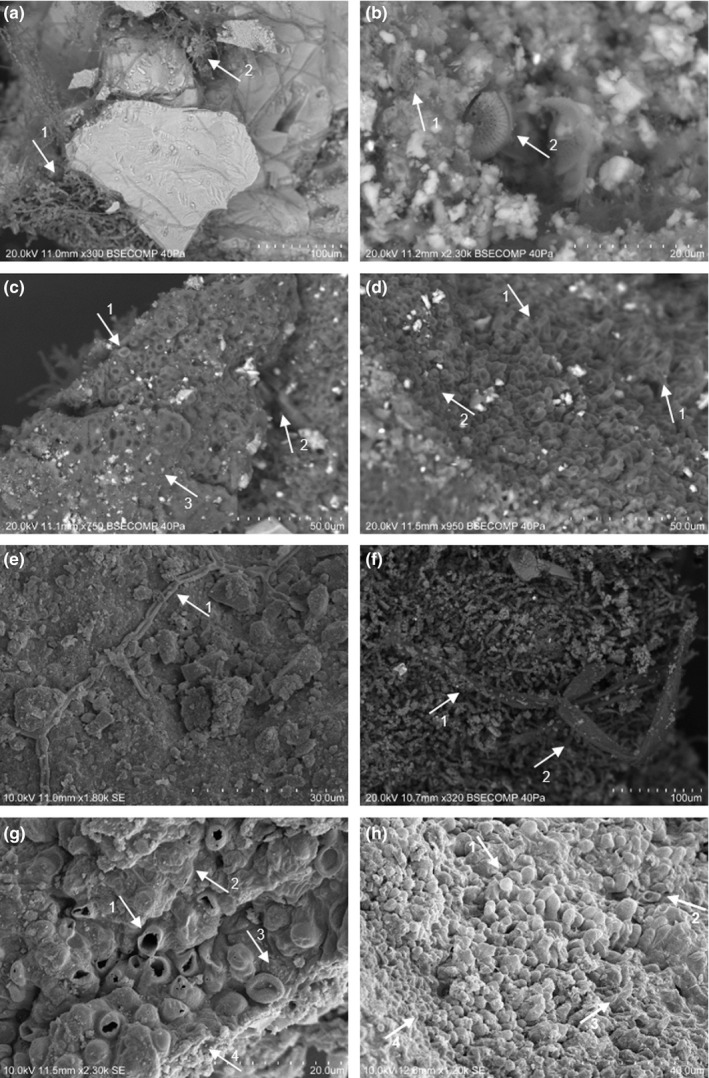
SEM micrographs showing microalgae (b, c, d, g, h), cyanobacteria (a, c, d) bacteria (c, d, g, h), and filamentous fungi (a, e, f) proliferating on stone materials (Legend; a1—cyanobacteria; a2—filamentous fungi; b1—bacteria; b2—diatom; c1—yeast; c2—unidentified filamentous structure; c3—microalgae biofilm; d1—microalgae; d2—yeast; e1, f1, and f2—filamentous fungi; g1—microalgae; g2—unknown biofilm; g3—yeast; g4—bacterial biofilm; H1—microalgae and/or cyanobacteria; h2—yeast; h3—cyanobacteria; h4—biofilm)

SEM‐EDX analysis enabled the detection of organic material on the surface of the stone (Figure 2a–b). It was possible to identify calcium (Ca), aluminum (Al), and silicon (Si) as the main constituents of the stone materials but also the concomitant presence of carbon (C), nitrogen (N), oxygen (O), and sulfur (S) on the stone surface which can be an indicative of the presence of biological material, being further confirmed by SEM analysis (Figure 3). The elemental characterization performed on the three different altered areas of the Convent revealed similar composition but distinct material morphology. In some cases (Figure 2b), the stone material was powdered, which seems to be related with the presence of microbial contaminants, namely through fungal proliferation, corroborated further by SEM (Figure 3). Biofilms (microbiological cells encased in an extracellular polymeric substance matrix) or more specifically subaerial biofilms (SABs: microbial communities at the stone–air interface) were frequently found covering the stone surfaces of the Convent (Figure 2a), mainly in altered areas by pigmented stains (Donlan, 2002; Villa, Stewart, Klapper, Jacob, & Cappitelli, 2016). These damages are extremely harmful for stone materials and can induce irreversible alterations. These complex matrices represent a generic mechanism for survival used by microorganisms which involves surface attachment and growth of heterogeneous cells, avoiding the loss of energy and nutrients. Their development can weaken the stone structure, allowing in‐depth proliferation and acting as a physical barrier against biocides, hindering mitigation treatments (Donlan, 2002; Kumar, Alam, Rani, Ehtesham, & Hasnain, 2017; Villa et al., 2016).

SEM micrographs showed (Figure 3) the presence of microalgae (including diatoms [Figure 3b]), cyanobacteria, bacteria, and filamentous fungi on stained areas. Furthermore, it is possible to observe hyphae proliferation between the stone structure and also detached fragments (Figure 3a) or powdered areas (Figure 3f). Microalgae, cyanobacteria, and bacteria are spread on all the sampled areas and seem to be associated with stained areas, which can be explained by the biofilms detected (Figure 3c,d,g,h; Goffredo et al., 2017; de la Torre, Gomez‐Alarcon, Vizcaino, & Garcia, 1992).

After the identification of microbiological proliferation, analyses for microflora characterization were performed. Using culture‐dependent methods, it was possible to identify the cultivable population present on these stone materials. This approach allowed the identification of 38 different isolates including fungi, yeasts, and bacteria (no cyanobacterial growth was detected in ASM‐1 and BG‐11 media). Regarding filamentous fungi, the most prevalent genera detected were *Cladosporium*, *Acremonium*, *Penicillium*, *Aspergillus,* and *Trichothecium.* Nevertheless, Gram‐positive coccus (*Arthrobacter* sp.), Gram‐negative coccus, *Bacillus* sp., and others unidentified bacteria were also detected on the pigmented areas.

*Arthrobacter* sp. and probably cyanobacteria (detected by SEM and NGS only classified at phylum level) seem to contribute for the appearance of stains. On the other hand, the presence of fungi of the genus *Penicillium* seems to be related with surface degradation and with the powdering processes that are visible on several areas of the Convent, particularly in the PC and the MC.

Until very recently, microbial identification required the isolation of pure cultures followed by multiple physiological and biochemical tests. However, the use of approaches aiming the isolation of cultivable microorganisms was a major gap in biodeterioration studies on monuments in the last decades (Saiz‐Jimenez, 2003; Seaward, 1997). Thankfully, nowadays HTS technology has allowed the analysis of significant diversity and microbiological community richness, revolutionizing the knowledge of the microbiological communities present in complex systems like cultural heritage assets (Chimienti et al., 2016; Cutler et al., 2013; Dias et al., 2018; Rosado et al., 2014; Tonon et al., 2019). In this work, prokaryote and eukaryote communities were assessed by NGS.

The microbiota thriving on these altered stone areas is quite diversified, being differently distributed: 381 bacterial and 17 fungal OTUs were obtained from the MC area; 112 bacterial and 88 fungal OTUs were obtained from the PC area; and 500 bacterial and 28 fungal OTUs were obtained from the Manueline Window (MW) area (data not shown). According to these results, the PC, the MC, and the MW areas are colonized mainly by bacterial communities.

**FIGURE 4:**
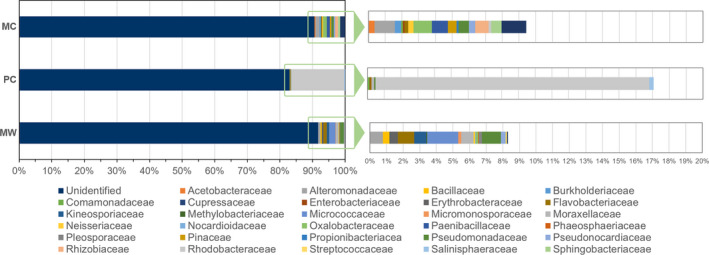
Predominant families of prokaryote population present on the Manueline Window (MW), the Primitive Cloister (PC), and the 1st floor of the Main Cloister (MC) of the Convent of Christ

**FIGURE 5:**
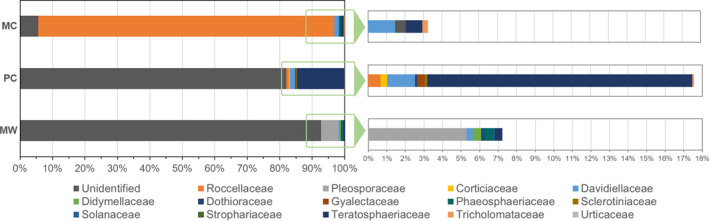
Predominant families of eukaryote population present on the Manueline Window (MW), the Primitive Cloister (PC), and the 1st floor of the Main Cloister (MC) of the Convent of Christ

The results show that the prokaryotic population present on the MW belongs to the following families: Alteromonadaceae (0.78%), Bacillaceae (0.39%), Flavobacteriaceae (1%), Micrococcaceae (1.85%), Moraxellaceae (0.75%), and Pseudomonadaceae (1.14%; Figure 4), while the prevalent fungal population (Figure 5) are Ascomycota fungi from the Pleosporaceae (5.28%), Davidiellaceae (0.34%), Didymellaceae (0.45%), Dothioraceae (0.17%), Phaeosphaericeae (0.51%), and Teratosphaeriaceae (0.40%) families.

Regarding the PC, the most representative prokaryotic communities (Figure 4) belong to the Rhodobacteraceae (16.32%), Salinisphaeraceae (0.24%), Comamonadaceae (0.12%), and Moraxellaceae (0.16%) families, whereas the predominant fungal population (Figure 5) are divided into Basidiomycota that belong to the family Corticiaceae (0.36%) and Ascomycota fungi from the Davidiellaceae (1.5%), Gyalectaceae (0.41%), Roccellaceae (0.67%), and Teratosphaeriaceae (14.25%) families.

Moreover, regarding the prokaryotic population the 1st floor of MC is dominated by Alteromonadaceae (1.21%), Oxalobacteraceae (1.11%), Paenibacillaceae (0.95%), Rhizobiaceae (0.79%), Sphingobacteriaceae (0.63%), and Xanthomonadaceae (1.48%) families (Figure 4), while the fungal population (Figure 5) predominantly belong to the Davidiellaceae (1.47%), Phaeosphaeriaceae (0.59%), Teratosphaeriaceae (0.88%), and Tricholomataceae (0.29%) families.

**FIGURE 6:**
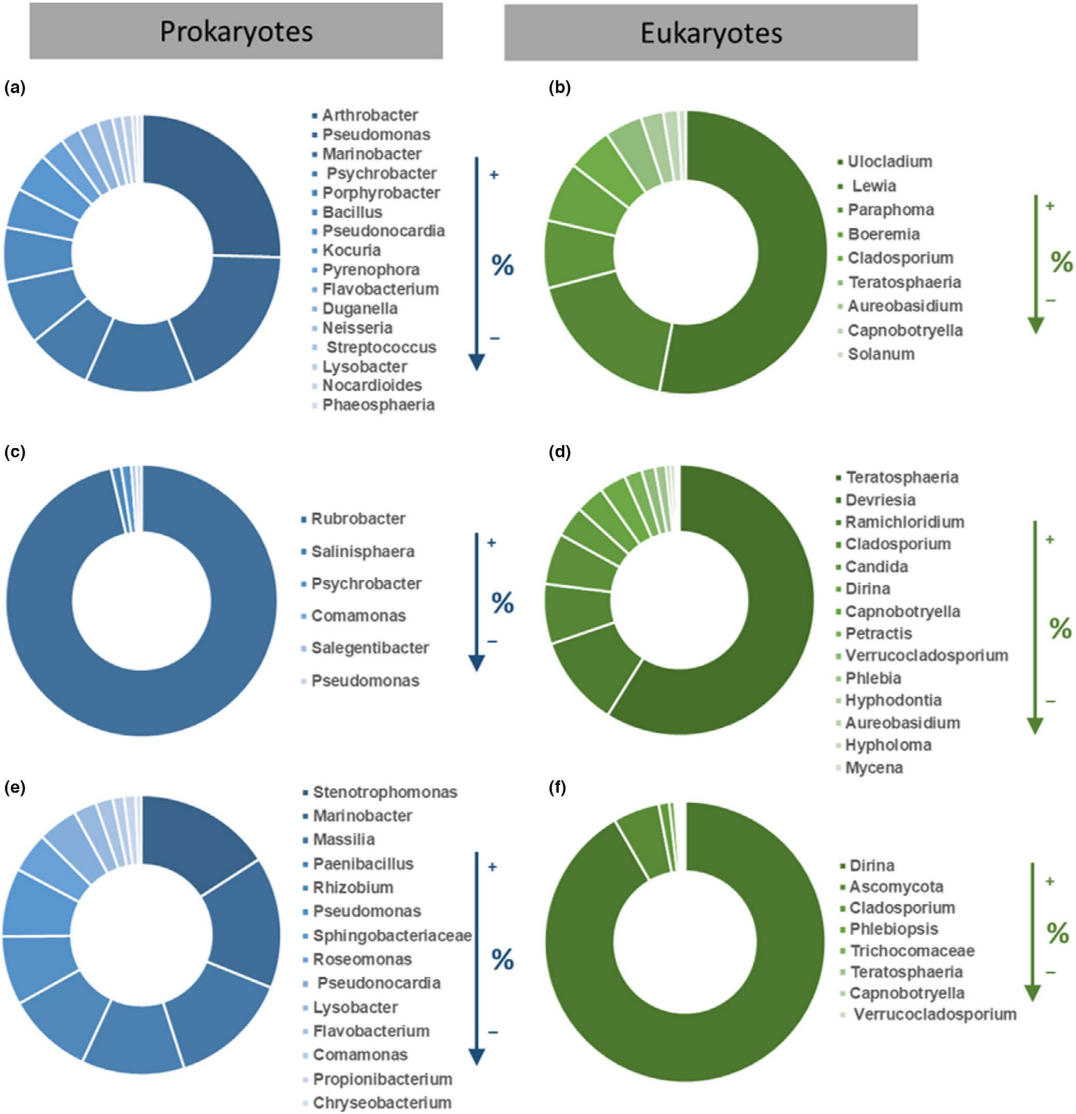
Relative abundance of prokaryote and eukaryote population present on the damaged areas of the Convent of Christ, identified at genus level (a, b—Manueline Window; c, d—Primitive Cloister; e, f—1st floor of the Main Cloister)

Additionally, a detailed analysis was performed at the genus level in order to characterize the relative abundance of prokaryote and eukaryote population present on the damaged areas of the Convent. These results are summarized in Figure 6.

The MW area is dominated by bacteria from the genera *Stenotrophomonas* (1.27%), *Marinobacter* (1.21%), *Massilia* (1.10%), *Paenibacillus* (0.95%), *Rhizobium* (0.79%), and *Roseomonas* (0.37%). However, more than 93% of the prokaryotic population remain unidentified at the genus level. On the other hand, the fungal population identified were the lichenized fungi from the genus *Dirina* (91%), followed by *Cladosporium* (1.18%), *Phlebiopsis* (0.59%), *Teratosphaeria* (0.29%), *Capnobotryella* (0.29%), and *Verrucocladosporium* (0.29%), remaining only 7% of this population unidentified at the genus level.

On the PC area, bacteria from the genus *Rubrobacter* (16.3%) were the most predominant microorganism identified, followed by the genera *Salinisphaera* (0.24%), *Psychobacter* (0.16%), *Comamonas* (0.12%), *Salegentibacter* (0.08%), and *Pseudomonas* (0.08%). Unfortunately, 83% of the prokaryotic population remained unidentified at the genus level. The presence of *Rubrobacter* sp. may contribute for the pink/orange stains (Chimienti et al., 2016; Gaylarde et al., 2017; Jurado, Miller, Alias‐Villegas, Laiz, & Saiz‐Jimenez, 2012; Laiz et al., 2009; Mihajlovski, Gabarre, Seyer, Bousta, & Martino, 2017) which are visible to the naked eye. Regarding the fungal population here present, fungi of the genus *Teratosphaeria* (11.24%) was the main microorganism identified. Fungi from the genera *Devriesia* (2.12%), *Ramichloridium* (1.40%), *Cladosporium* (1.19%), *Dirina* (0.67%), *Capnobotryella* (0.62%), *Petractis* (0.41%), *Verrucocladosporium* (0.31%), *Phlebia* (0.26%), *Teratosphaeria* (0.26%), *Aureobasidium* (0.10%), *Hyphodontia* (0.10%), *Hypholoma* (0.05%), and *Mycena* (0.05%) were also identified. In this area, almost 83% of the eukaryotic population does not match with any known sequence.

In the case of the 1st floor of the MC, 96% of the bacterial population remained unknown. Besides this, it was possible to identify bacteria from the genera *Arthrobacter* (1.57%), *Pseudomonas* (1.03%), *Marinobacter* (0.78%), *Bacillus* (0.39%), *Kocuria* (0.29%), *Acinetobacter* (0.29%), *Pseudonocardia* (0.29%), *Duganella* (0.14%), *Streptococcus* (0.07%), *Lysobacter* (0.07%), and *Nocardioides* (0.04%). Similarly, the fungal population of the 1st floor of the MC remains unidentified (93%), and it was only possible to characterize some fungi at the genera: *Ulocladium* (3.52%), *Lewia* (1.19%), *Paraphoma* (0.51%), *Boeremia* (0.45%), *Cladosporium* (0.34%), *Teratosphaeria* (0.28%), *Aureobasidium* (0.17%), *Capnobotryella* (0.11%), and *Solanum* (0.06%).

This metagenomic analysis provides an enhanced resolution up to the genus level and their relative abundance on the different areas analyzed, corroborating the results of SEM analyses and culture‐dependent techniques.

Noteworthy is the presence of *Rubrobacter* sp., *Arthrobacter* sp., *Roseomonas* sp., *and Marinobacter* sp. on stained areas that seems to be associated to stone color alteration (Chimienti et al., 2016; Gaylarde et al., 2017; Jurado et al., 2012; Laiz et al., 2009; Mihajlovski et al., 2017), while *Ulocladium* sp.*, Cladosporium* sp., *and Dirina* sp. are present on the structurally damaged and powdered areas (Dias et al., 2018; Ljaljević‐Grbić & Vukojević, 2009; Polo et al., 2012; Salvadori & Municchia, 2016).

The presence of the lichen *Dirina* sp. and its destructive effect has been observed and reported on Italian, Spanish, and Portuguese monuments during the last two decades. Notwithstanding lichens being usually regarded as major biodeterioration agents of geological materials, some studies have indicated strong evidence that environmental changes have been contributing for the increasingly detrimental invasion of these aggressive organisms. The *Dirina* sp. lichen is widespread throughout many areas of Europe, probably due to its reproductive strategy, its ability to exploit man‐made substrata, and the absence of other competing species resulting from the increasing air pollution (Coutinho et al., 2013; Edwards, 2007; Pinna, 2014; Seaward, 1997; Tonon et al., 2019; Zagari, Antonelli, & Urzi, 2000).

Thus, advances in high‐throughput analysis have provided new tools for the identification of microorganisms present in complex systems, which can help in the studies of cultural heritage field in order to define strategies to protect the assets from biocolonization and develop approaches to safeguard the monuments, being crucial the implementation of NGS technology in CHs workflows to fully characterize the microbiota.

Despite the fact that metagenomic analyses provide information about the genomic content of microorganisms (living or not), other approaches like metatranscriptomics and metaproteomics can be used to gain information on the active portion of the microbiota that thrive on stone monuments.

The whole perception of the biocolonizers and their effects enables the development of better intervention approaches to conduct the preservation of stone structures, prevent the loss of their aesthetic and historical value and structural integrity, benefiting both cultural and economic aspects (Aalil et al., 2016).

A long‐term monitoring is ongoing in the Convent of Christ, encompassing in situ measurements of temperature and relative humidity, microanalytical analyses to detect additional alterations and assessment of biocolonizers changes, according to the yearly seasons.

Cleaning and preventing actions based on natural biocompounds, whose efficacy and effectiveness were already tested by our research group, will be considered to eliminate, control, and prevent microorganisms’ development (Rosado et al., 2019) in the near future. Moreover, a screening of the microbial communities before and after the application of biocides as well as their monitoring throughout time is clearly needed, and this should be carried out in the framework of cultural heritage safeguard.

## 4 CONCLUSIONS

The Convent of Christ is a great example of an important stone building that has suffered significant alterations apparently caused by biocolonization, providing an ecological niche for the survival of microorganisms like bacteria, yeast, fungi, and lichens.

The aesthetic damages throughout the stone of the Convent, such as stains and biofilms, seem to be induced by bacteria, mainly by the strains *Rubrobacter* sp., *Arthrobacter* sp., *Roseomonas* sp., *and Marinobacter* sp, while fungi (*Ulocladium* sp.*, Cladosporium* sp.) and lichens (*Dirina* sp.) appear to be the main responsible for structural alterations.

Among the techniques (CDM, SEM, NGS) used for monitoring the microbiological agents that cause biodeterioration of cultural heritage assets, NGS is currently the most promising approach. With this innovative application, we hope to contribute, as much as possible, to the knowledge of the microbial population that are colonizing stone materials as well as to understand its effect on stone decay.

We believe that this study is a step forward for the implementation of an effective and accurate plan of conservation and intervention (including mitigation treatment) of a UNESCO World Heritage monument, the Convent of Christ, to avoid the destruction of this cultural asset and to promote its preservation and safeguard.

## CONFLICT OF INTEREST

None declared.

## AUTHOR CONTRIBUTIONS

Tânia Rosado supported in conceptualization, investigation, and writing, reviewing, and editing of the manuscript; equally contributed to formal analysis; and took lead in writing the original draft of the manuscript. Luís Dias equally contributed to formal analysis and supported in investigation and writing the original draft of the manuscript. Mónica Lança, Carla Nogueira, and Rita Santos supported in formal analysis. M Rosário Martins equally contributed to investigation and supported in writing, reviewing, and editing of the manuscript. António Candeias and José Mirão supported in funding acquisition and writing, reviewing, and editing of the manuscript, and equally contributed to investigation. Ana Teresa Caldeira took lead in conceptualization, funding acquisition, project administration, and supervision; equally contributed to investigation; and supported in writing, reviewing, and editing of the manuscript.

## ETHICS STATEMENT

None required.

## Data Availability

All data generated or analyzed during this study are provided in full in the results section of this paper. Raw data sets from NGS results are available in Appendix.

## APPENDIX A

**TABLE A1:** Taxonomic identification of eukaryotic population present on Manueline Window (MW)

# Classification									
Domain	Kingdom	Phylum	Class	Order	Family	Genus	Species	% hits	Num hits
Eukaryota	Fungi							89.94%	1583
Eukaryota	Fungi	Ascomycota	Dothideomycetes	Pleosporales	Pleosporaceae	Ulocladium		3.52%	62
Eukaryota	Fungi	Ascomycota	Dothideomycetes	Pleosporales				1.48%	26
Eukaryota	Fungi	Ascomycota						1.31%	23
Eukaryota	Fungi	Ascomycota	Dothideomycetes	Pleosporales	Pleosporaceae	Lewia	*L. infectoria*	1.19%	21
Eukaryota	Fungi	Ascomycota	Dothideomycetes	Pleosporales	Pleosporaceae			0.57%	10
Eukaryota	Fungi	Ascomycota	Dothideomycetes	Pleosporales	Phaeosphaeriaceae	Paraphoma		0.51%	9
Eukaryota	Fungi	Ascomycota	Dothideomycetes	Pleosporales	Didymellaceae	Boeremia	*B. exigua*	0.45%	8
Eukaryota	Fungi	Ascomycota	Dothideomycetes	Capnodiales	Davidiellaceae	Cladosporium		0.34%	6
Eukaryota	Fungi	Ascomycota	Dothideomycetes	Capnodiales	Teratosphaeriaceae	Teratosphaeria	*T. knoxdaviesii*	0.28%	5
Eukaryota	Fungi	Ascomycota	Dothideomycetes	Dothideales	Dothioraceae	Aureobasidium		0.17%	3
Eukaryota	Fungi	Ascomycota	Dothideomycetes	Capnodiales	Teratosphaeriaceae	Capnobotryella		0.11%	2
Eukaryota	Fungi	Ascomycota	Sordariomycetes	Hypocreales				0.06%	1
Eukaryota	Viridiplantae	Streptophyta	lamiids	Solanales	Solanaceae	Solanum		0.06%	1
Unidentified								98.07%	1726

**TABLE A2:** Taxonomic identification of prokaryotic population present on Manueline Window (MW)

# Classification									
Domain	Kingdom	Phylum	Class	Order	Family	Genus	Species	% hits	Num hits
Prokaryotes	Bacteria							81.11%	2275
Prokaryotes	Bacteria	Actinobacteria						4.46%	125
Eukaryota	Metazoa	Chordata	Mammalia	Primates	Hominidae	Homo	*H. sapiens*	1.93%	54
Eukaryota								1.82%	51
Prokaryotes	Bacteria	Actinobacteria	Actinobacteria	Actinomycetales	Micrococcaceae	Arthrobacter	*A. monumenti*	1.57%	44
Prokaryotes	Bacteria	Proteobacteria	Gammaproteobacteria	Pseudomonadales	Pseudomonadaceae	Pseudomonas	(Pseudomonas)	1.03%	29
Prokaryotes	Bacteria	Bacteroidetes	Flavobacteriia	Flavobacteriales	Flavobacteriaceae			0.86%	24
Prokaryotes	Bacteria	Proteobacteria	Gammaproteobacteria	Alteromonadales	Alteromonadaceae	Marinobacter		0.78%	22
Prokaryotes	Bacteria	Actinobacteria	Actinobacteria	Actinomycetales	Kineosporiaceae			0.71%	20
Prokaryotes	Bacteria	Proteobacteria	Alphaproteobacteria					0.61%	17
Prokaryotes	Bacteria	Proteobacteria	Gammaproteobacteria					0.61%	17
Prokaryotes	Bacteria	Proteobacteria	Alphaproteobacteria	Sphingomonadales	Erythrobacteraceae	Porphyrobacter		0.46%	13
Prokaryotes	Bacteria	Proteobacteria	Gammaproteobacteria	Pseudomonadales	Moraxellaceae	Psychrobacter		0.46%	13
Prokaryotes	Bacteria	Firmicutes	Bacilli	Bacillales	Bacillaceae	Bacillus		0.39%	11
Prokaryotes	Bacteria	Proteobacteria						0.32%	9
Prokaryotes	Bacteria	Actinobacteria	Actinobacteria	Actinomycetales				0.29%	8
Prokaryotes	Bacteria	Actinobacteria	Actinobacteria	Actinomycetales	Micrococcaceae	Kocuria		0.29%	8
Prokaryotes	Bacteria	Proteobacteria	Gammaproteobacteria	Pseudomonadales	Moraxellaceae	Acinetobacter		0.29%	8
Prokaryotes	Bacteria	Actinobacteria	Actinobacteria	Actinomycetales	Pseudonocardiaceae	Pseudonocardia		0.29%	8
Prokaryotes	Bacteria	Cyanobacteria						0.21%	6
Prokaryotes	Bacteria	Proteobacteria	Betaproteobacteria					0.18%	5
Eukaryota	Fungi	Ascomycota	Dothideomycetes	Pleosporales	Pleosporaceae	Pyrenophora	*P. tritici‐repentis*	0.18%	5
Prokaryotes	Bacteria	Actinobacteria	Actinobacteria	Actinomycetales	Micromonosporaceae			0.18%	5
Prokaryotes	Bacteria	Proteobacteria	Betaproteobacteria	Burkholderiales	Oxalobacteraceae	Duganella		0.14%	4
Prokaryotes	Bacteria	Bacteroidetes	Flavobacteriia	Flavobacteriales	Flavobacteriaceae	Flavobacterium		0.14%	4
Prokaryotes	Bacteria	Fusobacteria						0.14%	4
Prokaryotes	Bacteria	Proteobacteria	Betaproteobacteria	Neisseriales	Neisseriaceae	Neisseria	*N. lactamica*	0.11%	3
Prokaryotes	Bacteria	Proteobacteria	Gammaproteobacteria	Pseudomonadales	Pseudomonadaceae	Pseudomonas	*P. tolaasii*	0.11%	3
Prokaryotes	Bacteria	Proteobacteria	Alphaproteobacteria	Rhizobiales	Methylobacteriaceae			0.07%	2
Prokaryotes	Bacteria	Firmicutes	Bacilli	Lactobacillales	Streptococcaceae	Streptococcus		0.07%	2
Prokaryotes	Bacteria	Proteobacteria	Gammaproteobacteria	Xanthomonadales	Xanthomonadaceae	Lysobacter		0.07%	2
Eukaryota	Fungi	Ascomycota	Dothideomycetes	Pleosporales	Phaeosphaeriaceae	Phaeosphaeria	*P. nodorum*	0.04%	1
Prokaryotes	Bacteria	Actinobacteria	Actinobacteria	Actinomycetales	Nocardioidaceae	Nocardioides		0.04%	1
Eukaryota	Viridiplantae	Streptophyta	Coniferopsida	Coniferales	Cupressaceae			0.04%	1
Prokaryotes	Bacteria	Proteobacteria	Alphaproteobacteria	Rhizobiales				0.04%	1
Unidentified								96.08%	2695

**TABLE A3:** Taxonomic identification of eukaryotic population present on Primitive Cloister (PC)

# Classification									
Domain	Kingdom	Phylum	Class	Order	Family	Genus	Species	% hits	Num hits
Eukaryota	Fungi							39.95%	771
Eukaryota	Fungi	Ascomycota						35.18%	679
Eukaryota	Fungi	Ascomycota	Dothideomycetes	Capnodiales	Teratosphaeriaceae	Teratosphaeria	*T. knoxdaviesii*	11.24%	217
Eukaryota	Fungi	Ascomycota	Dothideomycetes	Capnodiales				4.61%	89
Eukaryota	Fungi	Ascomycota	Dothideomycetes	Capnodiales	Teratosphaeriaceae	Devriesia	*D. hilliana*	2.12%	41
Eukaryota	Fungi	Ascomycota	Dothideomycetes	Capnodiales	mitosporic_Capnodia	Ramichloridium		1.40%	27
Eukaryota	Fungi	Ascomycota	Dothideomycetes	Capnodiales	Davidiellaceae	Cladosporium		1.19%	23
Eukaryota	Fungi	Ascomycota	Saccharomycetes	Saccharomycetales	mitosporic_Saccharo	Candida	*C. tropicalis*	0.73%	14
Eukaryota	Fungi	Ascomycota	Arthoniomycetes	Arthoniales	Roccellaceae	Dirina	*D. ceratoniae*	0.67%	13
Eukaryota	Fungi	Ascomycota	Dothideomycetes	Capnodiales	Teratosphaeriaceae	Capnobotryella		0.62%	12
Eukaryota	Fungi	Ascomycota	Lecanoromycetes	Ostropales	Gyalectaceae	Petractis	*P. nodispora*	0.41%	8
Eukaryota	Fungi	Basidiomycota						0.31%	6
Eukaryota	Fungi	Ascomycota	Dothideomycetes	Capnodiales	Davidiellaceae	Verrucocladosporium	*V. dirinae*	0.31%	6
Eukaryota	Fungi	Basidiomycota	Agaricomycetes	Corticiales	Corticiaceae	Phlebia	*P. aurea*	0.26%	5
Eukaryota	Fungi	Ascomycota	Dothideomycetes	Capnodiales	Teratosphaeriaceae	Teratosphaeria	*T. microspora*	0.26%	5
Eukaryota	Fungi	Ascomycota	Dothideomycetes	Dothideales	Dothioraceae	Aureobasidium		0.10%	2
Eukaryota	Fungi	Basidiomycota	Agaricomycetes	Agaricales				0.10%	2
Eukaryota	Fungi	Basidiomycota	Agaricomycetes	Corticiales	Corticiaceae	Hyphodontia		0.10%	2
Eukaryota	Fungi	Basidiomycota	Agaricomycetes					0.10%	2
Eukaryota	Fungi	Ascomycota	Leotiomycetes	Helotiales	Sclerotiniaceae	Botryotinia		0.10%	2
Eukaryota	Fungi	Basidiomycota	Agaricomycetes	Agaricales	Strophariaceae	Hypholoma	*H. fasciculare*	0.05%	1
Eukaryota	Fungi	Ascomycota	Sordariomycetes					0.05%	1
Eukaryota	Fungi	Basidiomycota	Agaricomycetes	Agaricales	Tricholomataceae	Mycena	*M. pura*	0.05%	1
Eukaryota	Viridiplantae	Streptophyta	fabids	Rosales	Urticaceae			0.05%	1
Unidentified								83.89%	1619

**TABLE A4:** Taxonomic identification of prokaryotic population present on Primitive Cloister (PC)

# Classification									
Domain	Kingdom	Phylum	Class	Order	Family	Genus	Species	% hits	Num hits
Prokaryotes	Bacteria	Actinobacteria						63.73%	1562
Prokaryotes	Bacteria							18.93%	464
Prokaryotes	Bacteria	Actinobacteria	Actinobacteria	Rubrobacterales	Rubrobacteraceae	Rubrobacter		16.32%	400
Prokaryotes	Bacteria	Proteobacteria	Gammaproteobacteria	Salinisphaerales	Salinisphaeraceae	Salinisphaera		0.24%	6
Prokaryotes	Bacteria	Proteobacteria	Gammaproteobacteria	Pseudomonadales	Moraxellaceae	Psychrobacter		0.16%	4
Prokaryotes	Bacteria	Proteobacteria	Betaproteobacteria	Burkholderiales	Comamonadaceae	Comamonas		0.12%	3
Eukaryota	Metazoa	Chordata	Mammalia	Primates	Hominidae	Homo	*H. sapiens*	0.12%	3
Prokaryotes	Bacteria	Bacteroidetes	Flavobacteriia	Flavobacteriales	Flavobacteriaceae	Salegentibacter		0.08%	2
Prokaryotes	Bacteria	Proteobacteria	Gammaproteobacteria	Pseudomonadales	Pseudomonadaceae	Pseudomonas		0.08%	2
Prokaryotes	Bacteria	Proteobacteria	Gammaproteobacteria					0.08%	2
Eukaryota								0.08%	2
Prokaryotes	Bacteria	Proteobacteria	Gammaproteobacteria	Enterobacteriales	Enterobacteriaceae			0.04%	1
Unidentified								99.88%	2448

**TABLE A5:** Taxonomic identification of eukaryotic population present on 1st Main Cloister (MC)

# Classification									
Domain	Kingdom	Phylum	Class	Order	Family	Genus	Species	% hits	Num hits
Eukaryota	Fungi	Ascomycota	Arthoniomycetes	Arthoniales	Roccellaceae	Dirina	*D. ceratoniae*	91.15%	309
Eukaryota	Fungi	Ascomycota						5.31%	18
Eukaryota	Fungi	Ascomycota	Dothideomycetes	Capnodiales	Davidiellaceae	Cladosporium		1.18%	4
Eukaryota	Fungi	Basidiomycota	Agaricomycetes	Polyporales	Phanerochaetaceae	Phlebiopsis	*P. gigantea*	0.59%	2
Eukaryota	Fungi	Ascomycota	Eurotiomycetes	Eurotiales	Trichocomaceae			0.29%	1
Eukaryota	Fungi	Ascomycota	Dothideomycetes	Capnodiales	Teratosphaeriaceae	Teratosphaeria	*T. knoxdaviesii*	0.29%	1
Eukaryota	Fungi	Ascomycota	Dothideomycetes	Capnodiales	Teratosphaeriaceae	Capnobotryella	(Capnobotryella)	0.29%	1
Eukaryota	Fungi	Ascomycota	Dothideomycetes	Capnodiales	Teratosphaeriaceae			0.29%	1
Eukaryota	Fungi	Ascomycota	Dothideomycetes	Capnodiales	Davidiellaceae	Verrucocladosporium	*V. dirinae*	0.29%	1
Eukaryota	Fungi							0.29%	1
Unidentified								7.67%	26

**TABLE A6:** Taxonomic identification of prokaryotic population present on 1st Main Cloister (MC)

# Classification									
Domain	Kingdom	Phylum	Class	Order	Family	Genus	Species	% hits	Num hits
Prokaryotes	Bacteria							72.57%	1376
Prokaryotes	Bacteria	Proteobacteria	Betaproteobacteria					9.34%	177
Prokaryotes	Bacteria	Proteobacteria	Gammaproteobacteria					3.43%	65
Eukaryota	Metazoa	Chordata	Mammalia	Primates	Hominidae	Homo	*H. sapiens*	2.27%	43
Prokaryotes	Bacteria	Proteobacteria	Gammaproteobacteria	Xanthomonadales	Xanthomonadaceae	Stenotrophomonas		1.27%	24
Prokaryotes	Bacteria	Proteobacteria	Gammaproteobacteria	Alteromonadales	Alteromonadaceae	Marinobacter		1.21%	23
Prokaryotes	Bacteria	Proteobacteria	Betaproteobacteria	Burkholderiales	Oxalobacteraceae	Massilia	*M. aerilata*	1.05%	20
Prokaryotes	Bacteria	Bacteroidetes						1.00%	19
Prokaryotes	Bacteria	Firmicutes	Bacilli	Bacillales	Paenibacillaceae	Paenibacillus		0.95%	18
Prokaryotes	Bacteria	Proteobacteria	Alphaproteobacteria	Rhizobiales	Rhizobiaceae	Rhizobium		0.79%	15
Prokaryotes	Bacteria	Proteobacteria	Gammaproteobacteria	Pseudomonadales	Pseudomonadaceae	Pseudomonas		0.63%	12
Prokaryotes	Bacteria	Bacteroidetes	Sphingobacteriia	Sphingobacteriales	Sphingobacteriaceae			0.63%	12
Eukaryota								0.53%	10
Eukaryota	Viridiplantae	Streptophyta	Coniferopsida	Coniferales	Pinaceae	Pinus		0.53%	10
Prokaryotes	Bacteria	Actinobacteria						0.47%	9
Prokaryotes	Bacteria	Proteobacteria	Alphaproteobacteria					0.37%	7
Prokaryotes	Bacteria	Proteobacteria	Alphaproteobacteria	Rhodospirillales	Acetobacteraceae	Roseomonas		0.37%	7
Prokaryotes	Bacteria	Cyanobacteria						0.37%	7
Prokaryotes	Bacteria	Proteobacteria	Betaproteobacteria	Burkholderiales	Burkholderiaceae	(Burkholderiaceae)		0.37%	7
Prokaryotes	Bacteria	Actinobacteria	Actinobacteria_(cla	Actinomycetales	Pseudonocardiaceae	Pseudonocardia		0.37%	7
Prokaryotes	Bacteria	Proteobacteria	Betaproteobacteria	Neisseriales	Neisseriaceae			0.32%	6
Prokaryotes	Bacteria	Proteobacteria	Gammaproteobacteria	Xanthomonadales	Xanthomonadaceae	Lysobacter		0.21%	4
Prokaryotes	Bacteria	Proteobacteria						0.16%	3
Prokaryotes	Bacteria	Proteobacteria	Alphaproteobacteria	Rhodobacterales	Rhodobacteraceae			0.16%	3
Prokaryotes	Bacteria	Bacteroidetes	Flavobacteriia	Flavobacteriales	Flavobacteriaceae	Flavobacterium		0.16%	3
Prokaryotes	Bacteria	Proteobacteria	Gammaproteobacteria	Enterobacteriales	Enterobacteriaceae			0.11%	2
Prokaryotes	Bacteria	Proteobacteria	Betaproteobacteria	Burkholderiales	Comamonadaceae	Comamonas	*C. koreensis*	0.11%	2
Prokaryotes	Bacteria	Actinobacteria	Actinobacteria	Actinomycetales	Propionibacteriacea	Propionibacterium	*P. acnes*	0.11%	2
Prokaryotes	Bacteria	Bacteroidetes	Flavobacteriia	Flavobacteriales	Flavobacteriaceae	Chryseobacterium		0.05%	1
Prokaryotes	Bacteria	Proteobacteria	Betaproteobacteria	Burkholderiales	Oxalobacteraceae	Massilia		0.05%	1
Eukaryota	Fungi	Ascomycota						0.05%	1
Unidentified								96.47%	1829
